# The efficiency and effectiveness of surgery information systems in Iran

**DOI:** 10.1186/s12911-020-01236-5

**Published:** 2020-09-16

**Authors:** Faezeh Abbasi, Reza Khajouei, Moghaddameh Mirzaee

**Affiliations:** 1 Department of Health Information Sciences, Faculty of Management and Medical Information Sciences, Kerman University of Medical Sciences, Haft-bagh Highway, PO Box: 7616911313, Kerman, Iran; 2 Medical Informatics Research Center, Institute for Futures Studies in Health, Kerman University of Medical Sciences, Kerman, Iran; 3 Department of Biostatistics and Epidemiology, School of Public Health, Kerman University of Medical Sciences, Kerman, Iran; 4 Modeling in Health Research Center, Institute for Futures Studies in Health, Kerman University of Medical Sciences, Kerman, Iran

**Keywords:** Evaluation, Efficiency, Effectiveness, Surgery information system

## Abstract

**Background:**

Despite the prevalent use and advantages of information systems in hospitals, some have failed to meet their predefined objectives. Surgery information system (SIS) is a sub-system of a hospital information system. Its effective and efficient operation could enhance patient care in the busy environment of operating rooms with multiple tasks. The objective of this study was to evaluate the effectiveness and efficiency of SIS in three educational hospitals.

**Methods:**

Data were collected using a questionnaire completed by 82 users of SIS. This questionnaire contains three parts: 1) participants’ demographic information, 2) questions regarding the efficiency of SIS, and 3) questions about its effectiveness. An independent sample t-test was used to compare the efficiency and effectiveness among systems. Chi-squared and Fisher tests were used to determine the relationship between the participants’ demographics and efficiency and effectiveness as well as the relationship between efficiency and effectiveness.

**Results:**

About 23% of the participants rated the system’s efficiency as low, 29% as medium, and 48% as high. Besides, 24% of the participants considered the effectiveness of the system as low, 31% as medium, and 45% as high. There was a significant correlation between the efficiency and effectiveness of SIS (*p* ≤ 0.0001).

**Conclusion:**

Based on the perspective of most participants (44%)the efficiency and effectiveness of both surgery information systems were acceptable. The results suggest that these systems should be designed in a way that facilitate user’s interaction and reduce the time takes to complete tasks. The results could be useful for developing and designing an efficient and effective system.

## Background

Hospital information systems (HIS) have been developed with the aim of collecting, storing, processing, and providing access to patients’ information through computers and communication tools. They are used for establishing communication among healthcare providers, managing patients’ information, and providing better services to patients [1]. An efficient and effective system is necessary to reach the goals of a health care organization. According to international standard organization (ISO), efficiency and effectiveness are two determinants of system acceptability. Effectiveness is the accurate and successful completion of the system’s goals by a user, and efficiency is the extent of effort and resources accurately and completely used to achieve these goals [2].

Many studies have shown [3–5] the efficiency and effectiveness of surgery information systems in reducing errors by improving legibility, completeness, and accuracy of information required for accurate decision making by physicians and doing their tasks.

Evidence shows that [6–9] lack of efficiency and effectiveness not only causes errors but also increases adverse effects and clinical risks for patients. Furthermore, the system will be inefficient, and clinical expenses will be incurred if the tasks are not completed on time. Therefore, an efficient and effective system can enhance patients’ care and safety.

Surgery information system (SIS) is a subsystem of a hospital information system that is used in operating rooms. Since the occurrence of errors in operating rooms is inevitable and has adverse consequences for patients, the surgery team needs an efficient and effective system to provide accurate and efficient information and improve patients care safety [10–12].

So far, no study has reported on the evaluation of the effectiveness and efficiency of surgery information systems. The majority of the studies conducted on surgery information systems have addressed topics such as the management of patient’s care [9], quality assurance, the management of waiting list in surgery information systems [13], the development of mobile-based surgery information system [11] and its architecture [14]. The aim of this study was to evaluate the effectiveness and efficiency of the surgery information system from the viewpoints of its users.

## Methods

This study was conducted in 3 hospitals affiliated to Kerman University of Medical Sciences (Shafa, Afzalipoor, Bahonar) in the fall of 2018. Currently, the Tirazheh surgery information system is used in two hospitals, and Peyvand Dadeh is used in the other hospital. Of the 136 users with active usernames and passwords, 82 use the system actively. All these 82 users were invited to participate in the study.

### Surgery information system

The surgery information systems evaluated in this study were the subsystems of two health information systems developed by Tirazheh and Peyvand Dadeh that are currently used in 236 and 67 educational hospitals in Iran, respectively. These systems have various parts, including patient profiles, patient lists, patient status, new surgeries, requests for services, blood requests, service records and audio records, service results, inpatient appointments, patient transfers, and delivery information. Using these systems, users can undertake activities such as transferring patients from the waiting lists to clinical wards and entering the information of services provided to the patient. Also, users can order services from other wards and see the results of the provided services.

### Data collection

The data was collected using a questionnaire that was developed by the researchers based on the review of usability questionnaires [15] of the system as well as a previous study [16]. This questionnaire was reviewed by three medical informaticians, one health information management specialist, and one operating room technician (Additional file 1). The questionnaire was modified based on the specialists’ comments and its content validity was confirmed. In designing this questionnaire, three questions from the previous study, and two questions from the usability questionnaire were used for the effectiveness section. as well as two questions from the previous study and two questions from the usability questionnaire were used for the efficiency section. In addition, twelve of the questions were designed by researchers. The reliability of the questionnaire was confirmed by calculating the internal consistency with a Cronbach’s alpha of 95%. The questionnaire was composed of three parts: 1) Demographic information such as sex, education, work experience, computer skills, the experience of working with a surgery information system, daily use of the surgery information system, and the experience of using the traditional system. 2) Specific questions for evaluating the effectiveness of the surgery information system (16 questions, one of which related to the overall effectiveness of the system). 3) Specific questions for evaluating the efficiency of the surgery information system (5 questions). We used a 9 point Likert scale (1 = the lowest level of agreement to 9 = the highest level of agreement) to answer the questions. Zero was assigned to the questions with no answer. Effectiveness was assessed based on user’s perceptions of achieving system goals while performing tasks. Efficiency was measured based on the users’ perceptions of reducing the time required to perform tasks.

Data were collected from the hospitals under study by the researcher after obtaining the required license issued by the research deputy of the faculty. Printed questionnaires were distributed by one of the researchers among the participants after explaining the purpose of the study. Verbal consent was obtained from the participants before the completion of the questionnaires. To ensure the completeness of all questionnaires, one of the researchers personally collected the questionnaires in the same day. In the case that the users did not have enough time to complete the questionnaires on the same day, the researcher visited the hospital the next day and asked them to cooperate.

### Data analysis

Data were analyzed using SPSS 22. We used independent sample t-test to compare the efficiency and effectiveness of the two systems. Chi-square test and Fisher test were used to investigate the relationship between demographic information and efficiency and effectiveness as well as to determine the relationship between the efficiency and effectiveness of the system. The anchors of the questionnaire were reduced to three categories for easy data analysis (0–3: low, 3.1–6: average, 6.1–9: high) [17]. Moreover, the efficiency and effectiveness of the surgery information system were considered as acceptable if the total score of more than 70% was obtained. Likewise, it was considered as marginal with a score between 50 and 70%, and as not acceptable with a score of less than 50% [18].

## Results

The demographic information of the participants is shown in Table 1. Of the 82 users who participated in the study, 85% were female and 92% had an academic education degree. Forty percent of the participants had 6–15 years of work experience. In addition, 60% of the participants had less than 5 years’ experience of working with a surgery information system, and 58% use the system for less than an hour per day. More than 81% of the users had the experience of working with the traditional system.

**Table 1:** Participants’ demographic information

Demographic information	N (%)
Gender	
Female	70 (85.4)
Male	12 (14.6)
Work experience (year)	
≥ 5	25 (31.6)
6–15	31 (39.2)
> 15	23 (29.1)
Education	
Non-academic	6 (7.4)
Academic	75 (92.6)
Work experience with computer (year)	
≥ 5	20 (33.3)
6–15	37 (61.7)
> 15	3 (5.0)
Computer skills (year)	
Elementary	24 (30.0)
Advanced	56 (70.0)
Experience of using surgery information system	
≥ 5	35 (60.3)
6–15	23 (39.7)
Daily use of surgery information system	
< 1	45 (57.7)
≥ 1	33 (42.3)
Experience of using traditional systems	
Yes	62 (80.5)
No	15 (19.5)

Table 2 shows the mean efficiency and effectiveness of the surgery information systems based on their vendors. In general, there was no significant difference between the efficiency and effectiveness of the surgery information systems between Tirazheh and Peyvand Dadeh (*p* > 0.05).

**Table 2:** Comparison of the mean efficiency and effectiveness of surgery information systems between the vendors

Vendor	Factor	Mean ± Std	*p*-value
Tirazheh	Efficiency	11.5 ± 1.4	0.3
Peyvand Dadeh		11.05 ± 2.8	
Tirazheh	Effectiveness	35.4 ± 8.9	0.9
Peyvand Dadeh		34.8 ± 8.6	

The rating of the efficiency and effectiveness of the surgery information systems based on demographic information of the participants is shown in Table 3. There was no meaningful correlation between any of the participants’ demographic information (gender, work experience, education, experience of working with computer, computer skills, experience of using a surgery information system, daily use of the surgery information system, and experience of using the traditional system) with efficiency and effectiveness of the surgery information systems (*p* > 0.05).

**Table 3:** The rating of efficiency and effectiveness of the systems based on demographic information of the participants

Demographic information	Variables	Effectiveness			Efficiency		
		Acceptable N(%)	Marginal N (%)	Not acceptable N(%)	Acceptable N(%)	Marginal N(%)	Not acceptable N(%)
**Gender**	female	32 (45.7)	31 (44.3)	7 (10)	41 (58.6)	19 (27.1)	10 (14.3)
	male	5 (41.7)	5 (41.7)	2 (16.7)	9 (75.0)	1 (8.3)	2 (16.7)
**Work experience (year)**	≥5	12 (48)	10 (40)	3 (12)	15 (60)	7 (28)	3 (12)
	6–15	16 (51.6)	12 (38.7)	3 (9.7)	19 (61.3)	7 (22.6)	5 (16)
	16<	8 (34.8)	12 (52.2)	3 (13)	14 (60.9)	5 (21.7)	4 (17.4)
**Education**	Academic	3 (50)	3 (50)	0 (0)	5 (83.3)	0 (0)	1 (16.7)
	Non-academic	34 (45.3)	32 (42.7)	9 (12)	44 (58.7)	20 (26.7)	11 (14.7)
**Experience of working with computers (year)**	≥5	10 (50)	7 (35)	3 (15)	14 (70)	2 (10)	4 (20)
	6–15	17 (45.9)	17 (45.9)	3 (8.1)	21 (56.8)	11 (29.7)	5 (13.5)
	16<	1 (33.3)	2 (66.7)	0 (0)	2 (66.7)	1 (33.3)	0 (0)
**Computer skills**	elementary	6 (25)	14 (58.3)	4 (16.7)	12 (50)	8 (33.3)	4 (16.7)
	advanced	29 (51.8)	22 (39.3)	5 (8.9)	36 (64.3)	12 (21.4)	8 (14.3)
**Experience of working with surgery information system (year)**	≥5	19 (54.3)	12 (34.3)	4 (11.4)	25 (71.4)	5 (14.3)	5 (14.3)
	6–15	12 (52.2)	10 (43.5)	1 (4.3)	14 (60.9)	7 (30.4)	2 (8.7)
**Daily use of surgery information system (hour)**	< 1	21 (46.7)	20 (44.4)	4 (8.9)	31 (68.9)	9 (20.0)	5 (11.1)
	1≤	16 (48.5)	14 (42.4)	3 (9.1)	18 (54.5)	10 (30.3)	5 (15.2)
**Experience of using the traditional system**	Yes	31 (50)	26 (41.9)	5 (8.1)	38 (61.3)	17 (27.4)	7 (11.3)
	No	4 (26.7)	9 (60)	2 (13.3)	9 (60)	3 (20)	3 (20)

In general, about 23% of the participants considered the efficiency of the surgery information systems as low, 29% considered it as medium, and 48% regarded it as high. Besides, 24% of the participants considered the system effectiveness as low, 31% considered it as medium and 45% regarded it as high.

In response to the overall question comparing the effectiveness of the surgery information system with the traditional system, 57% (*n* = 47) of the participants believed the surgery information system is far more effective. In addition, 45% (*n* = 37) of them rated the learnability of the system in a short period at a medium level. The results also showed that 56% (*n* = 46) of the participants believed that the surgery information system can increase the quality of documents and the confidentiality of information. Likewise, 51% believed that using the system would increase the accuracy in recording the information and save the users time (Table 4).

**Table 4:** The viewpoints of the participants concerning the efficiency and effectiveness of surgery information system

Factors related to the efficiency and effectiveness of the system	Low N(%)	Medium N(%)	High N(%)
***Effectiveness***			
More effective than the traditional system	17 (20.7)	18 (22.0)	47 (57.3)
No need to learn the system	37 (45.1)	21 (25.6)	24 (29.3)
Meeting the users’ needs	20 (24.4)	32 (39.0)	30 (36.6)
System ease of use	25 (30.5)	28 (34.1)	29 (35.4)
Reducing physicians’ error	18 (22.0)	22 (26.8)	39 (47.6)
Improving the safety of patients in operating room	27 (32.9)	28 (34.1)	27 (32.9)
Increasing patients’ satisfaction	27 (32.9)	30 (36.6)	24 (29.3)
Increasing the quality of the documents	14 (17.1)	21 (25.6)	46 (56.1)
Increasing the confidentiality of information	15 (18.3)	20 (24.4)	46 (56.1)
Easy documentation of reports	18 (22.0)	25 (30.5)	39 (47.6)
Increasing the reliability of information and data in patient records	13 (15.9)	27 (32.9)	42 (51.2)
Continuing patients care	16 (19.5)	30 (36.6)	36 (43.9)
Increasing the quality of care provided to patients	23 (28.0)	25 (30.5)	34 (41.5)
Increasing the accuracy of recording patients information	18 (22.0)	22 (26.8)	42 (51.2)
Facilitating communication among specialists	18 (22.0)	31 (37.8)	33 (40.2)
Facilitating communication among clinical wards s	12 (14.6)	24 (29.3)	45 (54.9)
**Efficiency**			
Learning of the system in a short period of time	19 (23.2)	37 (45.1)	25 (30.5)
Facilitating reporting	16 (19.5)	19 (23.2)	46 (56.1)
Saving time	22 (26.8)	18 (22.0)	42 (51.2)
Increasing the completion of documents	19 (23.2)	20 (24.4)	42 (51.2)
Reducing the duration of requests from other wards	16 (19.5)	23 (28.0)	42 (51.2)

According to the perspective of 44% of the participants who believed that the efficiency of the system was not acceptable and 22% of those who rated it as marginal and 10% of those who considered it as acceptable, the effectiveness of the system was also the same. There was a significant relationship between the efficiency and effectiveness of the surgery information system (*p* ≤ 0.0001) (Table 5).

**Table 5:** The relationship between the efficiency and effectiveness of surgery information system

Effectiveness				
**Efficiency**		Acceptable N(%)	Marginal N(%)	Not Acceptable N(%)
	Acceptable N(%)	36 (43.9)	14 (17.0)	0 (0)
	Marginal N(%)	1 (1.2)	18 (21.9)	1 (1.2)
	Not Acceptable N(%	0 (0)	4 (4.8)	8 (9.7)

## Discussion

### Principal findings

According to the perspectives 44% of participants in this study, the efficiency and effectiveness of the surgery information system were acceptable. Also, there was no significant difference between the efficiency and effectiveness of the surgery information system in the two companies. About 30% of users reported that they can learn how to use the system in a short period of time, though they need some training to use it successfully. Moreover, more than 50% of them claimed that using this system can improve the confidentiality and accuracy of patient’s information as well as the completion of documents. The findings of the present study showed that the users irrespective of their age, sex, education, experience of working with computers, computer skills, and experience of working with surgery information systems have the same viewpoint about the efficiency and effectiveness of the system.

In our study, 44.7% of the participants rated the system’s effectiveness as high and 48.5%of them rated its efficiency as high, as well as 44% of participants believed that the system effectiveness and efficiency were acceptable. In this regard, Meulendijk [19] evaluated a decision support system and showed that users performed their tasks better using the system compared to traditional method, but they sometimes made mistakes when working with the system. Furthermore, due to the users’ unfamiliarity with the user interface, it took more time to get tasks done by the system, leading to low efficiency. Hence, participants indicated that the system was marginally acceptable. Findings of a review study [20] that evaluated three aspects (effectiveness, efficiency, and satisfaction) of the clinical decision making support system showed a positive effect of this system on efficiency and effectiveness. Also, Treadwell study [21] assessed the usability of the personal digital assistant system for clinical examinations. According to the results, the effectiveness of this system was not different in the effectiveness of the paper-based system, but its efficiency was higher. Farrahi in a study [22] measuring the relationship between user interface problems of an admission, discharge, and transfer (ADT) module with usability attributes, found that the efficiency and effectiveness of that system were unacceptable. This was due to the user interface issues such as unclear function of some keys, lack of “help” option, and the existence of hidden menus in the system. These issues prevented a successful interaction of the users with system wasted their time. Findings of Sheehan study [23] also indicated that quality of a system interface can influence the duration of work and consequently the system performance. Moreover Guo study [24] showed that system interface problems reduce the efficiency because users have to devote more time to fulfill a task.

The results of the study showed a significant relationship between the efficiency and effectiveness of surgery information systems. The findings of the present study are in line with the results of the study conducted by Erik [25] concerning the relationship between the three usability attributes (efficiency, effectiveness, and satisfaction). Findings of his study also showed a meaningful correlation between the efficiency and effectiveness attributes. Ramadan [26], in a study evaluating the usability of geographic information system (GIS), showed that although the effectiveness score was higher than the efficiency score, consistent with our results, there was a significant relationship between these two attributes. In a study conducted by Georgsson [2] participants who did their tasks successfully were also quicker in performing them.

In our study, 51% of the participants evaluated high rate of the use of the surgery information system to increasing the precision of recording patients’ information. Increased accuracy in data recording reduces errors and re-work, which can decrease the time required to perform tasks and improve the system efficiency. Consistent with this finding, Maat study findings [5] showed that after performing the computerized physician order entry (CPOE) in the intensive care unit (ICU), the glucose calculation accuracy increased. Use of this system, in comparison with the manual computation, reduced the computation time.

In the present study, about 48% of the users indicated that the physicians’ errors reduced highly when using the system. Also, 34% of the participants believed that this system improved patient safety up to a medium level This finding is consistent with the results of a study by Hsieh [27] in which the use of mobile electronic medication administration record (ME-MAR) system reduced medication errors by nurses and consequently increased patient safety. About 37% of our study participants mentioned that this system could improve patient satisfaction at a moderate level. This finding is congruent with the results of Yilmaz’s study that investigated the use of an architectural system for the development of smart information systems [28].

Based on the results, about 35% of the participants rated the system’s ease of use as high. Since system ease of use saves the time users spend on the learning and using a system, it can affect the efficiency of that system. Therefore, the design of such systems should be simplified in their development phase to improve their efficiency. In this regard, a study conducted by Lee [29] regarding the efficiency of the nursing care planning system showed that ease of use of the system improved efficiency and satisfaction of users. Furthermore, the study by Khajouei [30] also showed that physicians were satisfied with CPOE ease of use and its effect on medication safety, workflow, and efficiency. Likewise, nurses also had a positive attitude towards it. In the present study, the 45%of the participants mentioned their need for training to use the system. This may not be the case for other systems. For example, Alnasser [31] in a study which investigated the usability of Twazon Arabic Weight Loss App showed that a large number of users did not need to learn the app before using it. The majority of participants in this study believed that the surgery information system improves communication among specialists (40%) and clinical wards (55%). Since the aim of this communication is to provide care for the patients, the facilitation of communication can improve patient care [32]. Sturzlinger [6], in a study concerning the efficiency and effectiveness of CPOE with integrated decision support systems, highlighted the improvement of communication and patient care after implementation of this CPOE. Georgiou [33] in a study revealed that the use of CPOE and clinical decision support system (CDSS) reduced the turnaround time for laboratory test results and improved patient care as a result.

Fifty-six percent of the participants in this study noted that surgery information systems increase the confidentiality of information. This finding is in line with the results of Dale’s study [34] on the investigation of the privacy, confidentiality, and security of information in electronic information systems. Participants in our study asserted that surgery information systems increase the speed of completing documents. This finding is consistent with the results of previous studies [9, 35].

### Implications of the study

Despite the existence of different studies on surgery information systems, no previous study has investigated the efficiency and effectiveness of this system. This research is the first study examining the efficiency and effectiveness of two surgery information systems used in Iranian hospitals. The methodology adopted in this study helped to investigate the opinions of real users concerning the efficiency and effectiveness of surgery information systems. Our findings suggest that policymakers, designers and developers of information systems select and develop efficient and effective systems that help users reach out the system’s goals such as increasing patient safety and satisfaction, reducing task completion time, improving accuracy of patient information and reducing errors rate.

### Limitations

This study had three limitations. First, the efficiency and effectiveness of the system were evaluated in three university hospitals. More accurate results can be obtained by choosing a bigger sample size form a broader environment. However, this research was carried out on two surgery information systems that are currently used in 303 university hospitals in Iran. Second, since the study was conducted on an already implemented system, it was not possible to compare the effectiveness and efficiency of the surgery information system with the traditional system. Yet, the findings of this study can be used by subsequent studies that plan to compare efficiency and effectiveness of the systems before and after implementation. Third, this study examined the efficiency and effectiveness of two surgery information systems (Tirazheh and Peyvand Dadeh). Although the surgery information systems developed by different vendors may have different efficiency and effectiveness based on each questionnaire component, due to the following reasons this may not have a major impact on our findings: a) the efficiency and effectiveness of these two systems were not significantly different, b) since these two systems have the same purpose and are designed for the same goal, they are similar in terms of interface features and capabilities and c) this study was conducted in three teaching hospitals affiliated with the same university, hence, they have almost similar staff composition, and the technical knowledge of their system users is similar.

## Conclusion

In the present study, the efficiency and effectiveness of the surgery information systems were acceptable. Also, the efficiency and effectiveness of the two surgery information systems in the two companies (Tirazheh and Peyvand Dadeh) were similar. Based on users’ perspectives, the selection and development of information systems should focus on the aspects that improve the successful interaction of the users with the systems and of the system and reduce the time required to perform tasks This study provided insights for healthcare policy-makers, and designers and developers of information systems to select and develop efficient and effective systems.

## Supplementary information

**Additional file 1.**

## Data Availability

The data generated and analyzed during this study are available from the corresponding author on reasonable request.

## Abbreviations

CPOE: Computerized physician order entry
HIS: Hospital information system
ISO: International standard organization
SIS: Surgery information system
GIS: Geographic information system
ME-MAR: Mobile electronic medication administration record
ADT: Admission, discharge, and transfer
ICU: Intensive Care Unit
EHR: Electronic Health record
CDSS: Clinical Decision Support System

## References

[1] Ahmadian L, Dorosti N, Khajouei R, Gohari SH (2017). Challenges of using hospital information systems by nurses: comparing academic and non-academic hospitals. Electron Physician.

[2] Georgsson M, Staggers N (2016). Quantifying usability: an evaluation of a diabetes mHealth system on effectiveness, efficiency, and satisfaction metrics with associated user characteristics. J Am Med Inform Assoc.

[3] Hardenbol AX, Knols B, Louws M, Askari M, Meulendijk M. Usability aspects of medication-related decision support systems in the outpatient setting: a systematic literature review. Health Informatics J. 2020;26(1):72–87. 10.1177/1460458218813732 30497336

[4] Chunmei R, Hualing H, Haihua Z (2018). Design and application of nursing CDSS based on structured EMR. Stud Health Technol Inform.

[5] Maat B, Rademaker CMA, Oostveen MI, Krediet TG, Egberts TCG, Bollen CW (2013). The effect of a computerized prescribing and calculating system on hypo- and hyperglycemias and on prescribing time efficiency in neonatal intensive care patients. JPEN J Parenter Enteral Nutr.

[6] Stürzlinger H, Hiebinger C, Pertl D, Traurig P (2009). Computerized Physician Order Entry - effectiveness and efficiency of electronic medication ordering with decision support systems. GMS Health Technol Assess.

[7] Nitrosi A, Bertolini M, Notari P, Botti A, Ginocchi V, Tondelli G (2013). Efficiency and effectiveness of an innovative RIS function for patient information reconciliation directly integrated with PACS. J Digit Imaging.

[8] Piva E, Sciacovelli L, Zaninotto M, Laposata M, Plebani M (2009). Evaluation of effectiveness of a computerized notification system for reporting critical values. Am J Clin Pathol.

[9] Hariyati RTS, Yani A, Eryando T, Hasibuan Z, Milanti A (2016). The effectiveness and efficiency of nursing care documentation using the SIMPRO model. Int J Nurs Knowl.

[10] Makary MA, Sexton JB, Freischlag JA, Millman EA, Pryor D, Holzmueller C (2006). Patient safety in surgery. Ann Surg.

[11] Shen-Yuan T, Chien-Liang C, Lun-Ping H (2015). Development of mobile-based intelligent surgery information system. 2015 11th international conference on heterogeneous networking for quality, reliability, Security and Robustness (QSHINE).

[12] Nouei MT, Kamyad AV, Soroush AR, Ghazalbash S (2015). A comprehensive operating room information system using the Kinect sensors and RFID. J Clin Monit Comput.

[13] Cristóvão R, Gomes P. Management of waiting list for surgery information system. In the European conference on information systems management. University of Ghent (p. 579). Belgium: Academic Conferences International Limited; 2011.

[14] Niiranen S, Välimäki A, Yli-Hietanen J (2008). Experiences from the architectural migration of a joint replacement surgery information system. Biomed Inform Insights.

[15] Garcia A (2013). UX research | standardized usability questionnaire.

[16] Sadoughi F, Aminpour F (2011). A review on the evaluation methods of health information systems. Iran J Med Educ.

[17] Ghazisaeedi M, Shahmoradi L, Ranjbar A, Sahraei Z, Tahmasebi F (2016). Designing a Mobile-based self-care application for patients with heart failure. J Health Biomed Inf.

[18] Bangor A, Kortum PT, Miller JT (2008). An empirical evaluation of the system usability scale. Int J Hum Comput Interact.

[19] Meulendijk MC, Spruit MR, Drenth-van Maanen AC, Numans ME, Brinkkemper S, Jansen PAF (2015). Computerized decision support improves medication review effectiveness: an experiment evaluating the STRIP Assistant's usability. Drugs Aging.

[20] Knols B, Louws M, Hardenbol A, Dehmeshki J, Askari M. The usability aspects of medication-related decision support systems in the inpatient setting: a systematic review. Health Informatics J. 2020;26(1):613–27. 10.1177/1460458219841167 31014159

[21] Treadwell I (2006). The usability of personal digital assistants (PDAs) for assessment of practical performance. Med Educ.

[22] Farrahi R, Rangraz Jeddi F, Nabovati E, Sadeqi Jabali M, Khajouei R (2019). The relationship between user interface problems of an admission, discharge and transfer module and usability features: a usability testing method. BMC Med Inform Decis Mak.

[23] Sheehan B, Lee Y, Rodriguez M, Tiase V, Schnall R (2012). A comparison of usability factors of four mobile devices for accessing healthcare information by adolescents. Appl Clin Inform.

[24] Guo J, Iribarren S, Kapsandoy S, Perri S, Staggers N (2011). Usability evaluation of an electronic medication administration record (eMAR) application. Appl Clin Inform.

[25] Frøkjær E, Hertzum M, Hornbæk K (2000). Measuring usability: are effectiveness, efficiency, and satisfaction really correlated? Proceedings of the SIGCHI conference on Human Factors in Computing Systems.

[26] Ben Ramadan AA, Jackson-Thompson J, Schmaltz CL (2017). Usability Assessment of the Missouri Cancer Registry's Published Interactive Mapping Reports: Round One. JMIR Hum Factors.

[27] Hsieh S-H, Hou IC, Cheng P-H, Tan C-T, Shen P-C, Hsu K-P (2010). Design and implementation of web-based mobile electronic medication administration record. J Med Syst.

[28] Yılmaz Ö, Erdur RC, Türksever M (2013). SAMS--a systems architecture for developing intelligent health information systems. J Med Syst.

[29] Lee T-T, Lee T-Y, Lin K-C, Chang P-C (2005). Factors affecting the use of nursing information systems in Taiwan. J Adv Nurs.

[30] Khajouei R, Wierenga PC, Hasman A, Jaspers MWM (2011). Clinicians satisfaction with CPOE ease of use and effect on clinicians' workflow, efficiency and medication safety. Int J Med Inform.

[31] Alnasser A, Kyle J, Alkhalifah A, Marais D (2018). Relationship between evidence requirements, user expectations, and actual experiences: usability evaluation of the Twazon Arabic weight loss app. JMIR Hum Factors.

[32] Kurahashi AM, Stinson JN, van Wyk M, Luca S, Jamieson T, Weinstein P (2018). The Perceived Ease of Use and Usefulness of Loop: Evaluation and Content Analysis of a Web-Based Clinical Collaboration System. JMIR Hum Factors.

[33] Georgiou A, Lang S, Rosenfeld D, Westbrook JI (2011). The use of computerized provider order entry to improve the effectiveness and efficiency of coagulation testing. Arch Pathol Lab Med.

[34] O’Brien DG, Yasnoff WA (1999). Privacy, confidentiality, and security in information systems of state health agencies. Am J Prev Med.

[35] Hsieh H-Y, Henker R, Ren D, Chien W-Y, Chang J-P, Chen L (2016). Improving effectiveness and satisfaction of an electronic charting system in Taiwan. Clin Nurse Spec.

