# Supplementary material for: The efficiency and effectiveness of surgery information systems in Iran
Source: BMC Med Inform Decis Mak. 2020 Sep 16;20:229. doi: 10.1186/s12911-020-01236-5 (PMC7493378; doi:10.1186/s12911-020-01236-5)
Supplement: Supplementary file 1 — Additional file 1. [file 12911_2020_1236_MOESM1_ESM.docx]

**Dear participant**

This questionnaire is designed to conduct a research entitled “The Efficiency and Effectiveness of Surgery Information Systems in Iran”. We would appreciate the time and effort you spend to answer the questions and thank your kind cooperation in advance. To fill this questionnaire, it’s not required to mention your name and the collected data will be analyzed collectively. Therefore, be assured that the provided answers will be treated in a strict confidence.

Reza Khajouei, Associate professor of Medical Informatics, Medical Informatics Research Center, Kerman University of Medical Sciences

Faeze Abbasi, MSc in Health Information Technology, Department of Health Information Sciences, Kerman University of Medical Sciences

1. Please provide the following demographic Information:

- Gender: Male: Female:
- Work experience (year)……………….
- Education: Lower than high school diploma High school diploma Associates or bachelor's degree Master's degree or higher

- Work experience with computers (year): ……………….
- Computer skills: Elementary Intermediate Advanced
- Duration of using surgery information system (month): ……………….
- Daily use of surgery information system: less than 1 hour 1 hour 2 hours more than 2 hours
- Have you ever used a traditional (paper-based) surgery information system?

Yes NO

1. Please specify the level of your agreement with each of the following statements about surgery information system by selecting the most appropriate number on a scale of 0-9 (0 = no opinion, 1 = lowest agreement and 9 = highest agreement).

| Disagree agree | | | | | | | | | No answer | Question |
| --- | --- | --- | --- | --- | --- | --- | --- | --- | --- | --- |
| 9 | 8 | 7 | 6 | 5 | 4 | 3 | 2 | 1 | 0 |  |
|  |  |  |  |  |  |  |  |  |  | This system is easy to use. |
|  |  |  |  |  |  |  |  |  |  | I do not need to learn the system. |
|  |  |  |  |  |  |  |  |  |  | Most users can learn how to use the system in a short period of time. |
|  |  |  |  |  |  |  |  |  |  | Using this system meets my needs. |
|  |  |  |  |  |  |  |  |  |  | Using this system is more effective than the traditional system (for example, less error in data entry) |
|  |  |  |  |  |  |  |  |  |  | Using this system reduces physicians' errors. |
|  |  |  |  |  |  |  |  |  |  | The use of this system improves patient safety in operating room. |
|  |  |  |  |  |  |  |  |  |  | This system increases patient satisfaction. |
|  |  |  |  |  |  |  |  |  |  | With this system, the quality of documents is increased. |
|  |  |  |  |  |  |  |  |  |  | Using this system facilitates reporting. |
|  |  |  |  |  |  |  |  |  |  | The use of this system increases the confidentiality of information. |
|  |  |  |  |  |  |  |  |  |  | Documentation of reports is easy with this system. |
|  |  |  |  |  |  |  |  |  |  | Using this system saves time. |
|  |  |  |  |  |  |  |  |  |  | Using this system increases the reliability of information and data in patient record. |
|  |  |  |  |  |  |  |  |  |  | Using this system increase the speed of documentation. |
|  |  |  |  |  |  |  |  |  |  | This system supports continuity of patient care. |
|  |  |  |  |  |  |  |  |  |  | This system, reduces the duration of requesting services from other wards. |
|  |  |  |  |  |  |  |  |  |  | This system increases the quality of healthcare services provided to patients. |
|  |  |  |  |  |  |  |  |  |  | This system increases the accuracy of patient information when recording. |
|  |  |  |  |  |  |  |  |  |  | Using this system facilitates communication among specialists. |
|  |  |  |  |  |  |  |  |  |  | Using this system facilitates communication among clinical wards. |
